# Performance of an artificial intelligence–based software in detecting pneumothorax on supine chest radiographs: a retrospective study

**DOI:** 10.1007/s10140-026-02448-4

**Published:** 2026-03-11

**Authors:** Hitomi Nakamura, Tomoki Wada, Ryota Inokuchi, Shouhei Hanaoka, Naoya Sakamoto, Kent Doi

**Affiliations:** 1 Department of Emergency and Critical Care Medicine, The University of Tokyo Hospital, 7-3-1 Hongo, Bunkyo-Ku, Tokyo, 113-0033 Japan; 2 Department of Radiology, The University of Tokyo Hospital, 7-3-1 Hongo, Bunkyo-Ku, Tokyo, 113-0033 Japan

**Keywords:** Artificial intelligence, Pneumothorax, Radiograph, Computed tomography

## Abstract

**Purpose:**

To evaluate the diagnostic performance of artificial intelligence (AI)-based software for pneumothorax detection on supine radiographs and its impact on physicians’ interpretation.

**Methods:**

This single-center retrospective study analyzed 114 hemithoraces with pneumothorax and 340 without, using computed tomography as the reference standard. We evaluated the performance of CXR-AID, an AI-based software, for pneumothorax detection on supine chest radiographs, and conducted a reader study to assess the utility of AI assistance. Sensitivities and specificities were adjusted for clustering within patients.

**Results:**

Sensitivity and specificity of the AI in detecting overall pneumothorax on a supine chest radiograph were 61.0% (95% confidence interval [CI], 50.6%–70.4%) and 94.3% (95% CI, 90.8%–96.5%), respectively. Sensitivity of the AI in detecting a large pneumothorax with a maximum radial interpleural distance > 35 mm was 97.4% (95% CI, 83.6%–99.6%). Sensitivity was significantly higher in the upper lung zone than in the lower lung zone (69.5% [95% CI, 59.3%–78.1%] vs. 37.5% [95% CI, 27.3%–48.8%]). In the reader study, the AI significantly improved resident sensitivity (46.8% to 57.3%, *P* < 0.001). For experts, the AI did not improve sensitivity significantly (*P* = 0.32) but significantly improved specificity (90.9% to 95.6%, *P* = 0.02).

**Conclusions:**

The AI demonstrated high sensitivity for detecting large pneumothoraces on supine radiographs, helping identify patients requiring tube thoracotomy. It may serve as a diagnostic safety net for residents by increasing sensitivity and enhances experts’ diagnostic confidence by improving specificity. However, pneumothorax detection in the lower lung zone remains challenging.

**Graphical Abstract:**

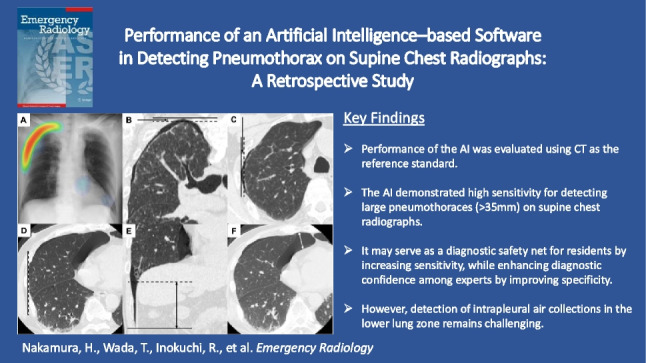

**Supplementary Information:**

The online version contains supplementary material available at 10.1007/s10140-026-02448-4.

## Background

Pneumothorax is a serious condition in critically ill patients admitted to emergency departments and intensive care units. Delayed diagnosis of pneumothorax can lead to patient deterioration, especially in those receiving mechanical ventilation [1]; hence, prompt detection of pneumothorax is required. Chest radiography is an initial imaging modality performed for the screening of pneumothorax [2–4]. However, critically ill patients usually can only tolerate chest radiography in the supine position. Previous reports have indicated that detection of pneumothorax using supine chest radiography remains challenging [5–9].

Artificial intelligence (AI) technology could aid physicians in detecting abnormalities in chest radiographs, including pneumothorax [10–15]. A related study reported that the performance of an AI-based system in detecting pneumothorax is poorer with supine X-rays than with upright X-rays [12]. However, whether the performance of AI-based software in detecting pneumothorax is affected by the size of the pneumothorax measured on computed tomography (CT) images remains unclear. Indeed, previous studies have demonstrated that a small pneumothorax (e.g., one with a maximum radial interpleural distance ≤ 35 mm on an axial CT image) can be managed conservatively [16, 17]. This indicates the importance of pneumothorax size in determining management strategies.

The present study aimed to evaluate the performance of a commercial AI-based software in detecting pneumothorax on supine chest radiographs, particularly for clinically significant air collections requiring tube thoracotomy as determined by CT measurements. Furthermore, we investigated the impact of the AI on the diagnostic performance of physicians.

## Methods

### Study setting and AI-based software

This single-center, retrospective study evaluated the performance of CXR-AID (Fujifilm, Tokyo, Japan) in detecting pneumothorax [18]. CXR-AID is a commercial AI-based software developed to detect pulmonary nodules, pulmonary consolidation, and pneumothorax on chest radiographs. When an abnormality is detected, the system displays a heatmap superimposed on the image using a spectral color scale (e.g., from blue for low probability to red for high probability). Notably, although the software detects the three distinct categories of findings, the generated heatmap does not specify the type of abnormality identified. Our institution has utilized this software in clinical practice since April 1, 2023.

### Data selection process

We identified CT scans performed at the University of Tokyo Hospital between April 1, 2023, and March 31, 2025, where radiology reports explicitly documented the presence or absence of pneumothorax. From this cohort, we included CT scans of patients who visited our emergency department or were admitted to our intensive care unit. Subsequently, we identified supine chest radiographs obtained within 6 h of the CT scans. If a patient had undergone multiple radiographic examinations within 6 h of a single CT scan, or vice versa, we selected a CT-radiograph pair for analysis based on the following criteria: matching chest tube status (both absent or both present) and the shortest time interval between the two examinations. Each right and left hemithorax was evaluated as a separate unit of analysis.

The size of the pneumothorax was assumed to be stable between the paired CT and radiography examinations. Consequently, hemithoraces that underwent tube thoracotomy between the paired examinations were excluded, as such a procedure would significantly alter the pneumothorax volume.

### Stand-alone AI performance

To evaluate the stand-alone diagnostic performance of the AI for pneumothorax detection, supine chest radiographs and their corresponding CT scans were independently reviewed by two board-certified specialists (an intensivist and a radiologist). Any discrepancies in the evaluation were resolved by consensus. CT scans were used as the reference standard to determine the presence, size, and location of pneumothorax. Additionally, concurrent findings were evaluated, including air-space opacification and radiological evidence of chronic lung diseases, specifically emphysema and interstitial lung disease.

**Fig. 1:**
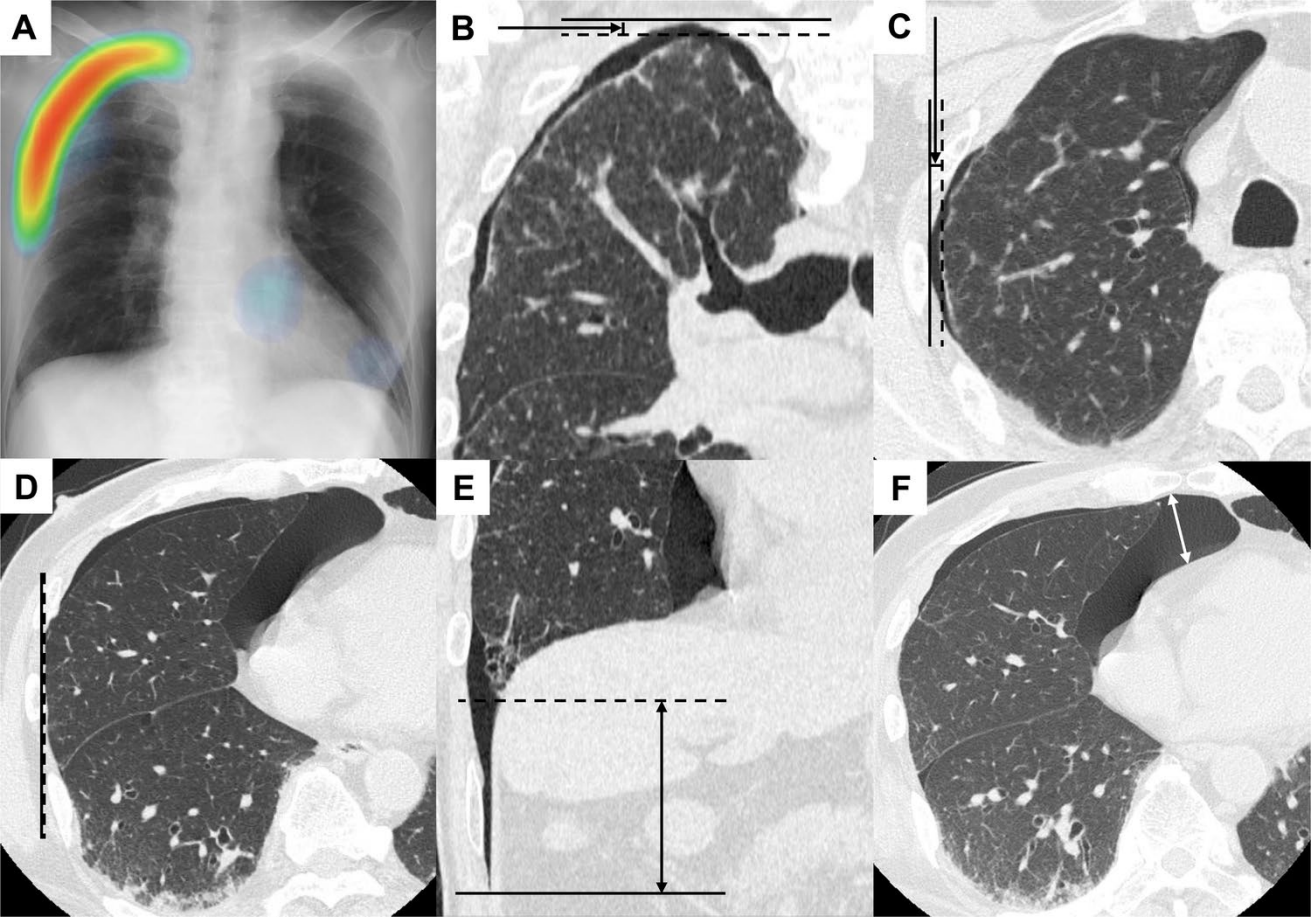
An example of a CXR-AID–generated heatmap for pneumothorax interpretation and measurement. (**A**) Supine chest radiograph with a CXR-AID–generated heatmap. The heatmap correctly highlighted an area with the intrapleural air collection in the right upper lung zone but missed the one in the right lower lung zone, when compared with the corresponding computed tomography (CT) images (B–F). (**B**) Coronally reconstructed CT image. The arrow indicates the apical interpleural distance (5 mm). (**C**) Axial CT image at the midpoint of the upper half of the lung. The arrow indicates the lateral interpleural distance (4 mm). (**D**) Axial CT image at the midpoint of the lower half of the lung showing a lateral interpleural distance of 0 mm. (**E**) Coronally reconstructed CT image. The double-headed arrow indicates the basal interpleural distance (64 mm). (**F**) Axial CT image showing the maximum interpleural radial distance of an air pocket (double-headed arrow, 32 mm). (B–E) Solid lines, parietal pleural edge; dotted lines, visceral pleural edge

On the supine chest radiographs, we evaluated the regions highlighted by the AI-generated heatmaps. Each heatmap was classified based on the CT reference standard as follows: true-positive, the heatmap correctly highlighted a region where intrapleural air was confirmed on CT; false-positive, the heatmap highlighted a region where no intrapleural air was present on CT, provided the specialists judged the highlighted region to represent a misinterpretation of a pneumothorax mimic; false-negative, the AI failed to generate a heatmap corresponding to a pneumothorax identified on CT; and true-negative, no heatmap suggesting a pneumothorax was generated in a region where no intrapleural air was present. When the AI highlighted a region suspected of intrapleural air, its anatomical location was categorized as being either superior or inferior to the hilum Fig. 1.

Radiographic quality was assessed based on patient rotation and X-ray beam angulation. Rotation was evaluated by the alignment of the thoracic spinous processes relative to the medial ends of the clavicles and was considered present if a spinous process overlapped with either medial clavicular end. Angulation was categorized by the position of the medial clavicular ends relative to the posterior ribs: cranial angulation was defined as the medial clavicular ends being superior to the third posterior rib, and caudal angulation was defined as being inferior to the fourth posterior rib.

### Pneumothorax measurement on CT images

The CT slice thickness and interval were set to 1 mm whenever possible; otherwise, the thinnest available setting was used. We measured the interpleural distances at several locations on the CT images of patients with pneumothorax Fig. 1. First, we measured three specific interpleural distances based on the Collins and Rhea methods to quantify pneumothorax size: (1) the apical distance; (2) the lateral distance at the midpoint of the upper half of the lung; and (3) the lateral distance at the midpoint of the lower half of the lung [19, 20]. Second, we measured the basal distance, defined as the distance from the deepest point of the costophrenic recess to the inferior margin of the lung parenchyma. This measurement was intended to serve as an indicator for intrapleural air collection in the lower lung zone, which forms the anatomical basis of the "deep sulcus sign" on supine radiographs [21, 22]. Finally, we measured the maximum radial distance of the largest air pocket in the axial image. This value corresponds to the “35-mm rule,” where a traumatic pneumothorax ≤ 35 mm in size is considered safe for observation without tube thoracotomy [16].

All measurements were performed using the built-in digital caliper tool of the SYNAPSE SAI viewer (Fujifilm, Tokyo, Japan), our institution's Picture Archiving and Communication System.

### Performance of physicians in unaided and AI-aided conditions

To evaluate the impact of the AI on physicians’ radiograph interpretation, a reader study was conducted involving six intensivists: three board-certified specialists (experts) and three residents. All physicians were blinded to clinical data and ground truth provided by corresponding CT scans. Original DICOM images were reviewed on high-resolution displays optimized for medical image visualization. The reader study followed a case-by-case sequential protocol. For each radiograph, physicians initially assessed the presence of pneumothorax on each side without AI assistance; immediately after recording the initial assessment, physicians reviewed the same radiograph with the AI-generated heatmaps and recorded their final diagnosis before proceeding to the next radiograph. Although the specific set of radiographs assigned to each physician varied, each individual dataset was enriched with a higher prevalence of pneumothorax compared to the overall study cohort. Within the total study dataset, each pneumothorax-positive radiograph was reviewed by two or three readers, whereas each negative radiograph was reviewed by one or two readers.

### Statistical analyses

In the evaluation of the stand-alone AI performance, radiological findings and pneumothorax size measurements were compared between pneumothoraces detected by the AI and those it missed. For the univariate analyses, continuous variables were compared using the clustered Wilcoxon rank-sum test, while categorical variables were analyzed using a logistic regression model within the generalized estimating equations (GEE) framework. Among the five measured interpleural distances, the two variables showing the lowest Spearman’s rank correlation coefficient were selected for the subsequent multivariable analysis. In addition, non-measurement variables with a* P*-value < 0.10 in the univariate analysis were included in the multivariable logistic regression model using GEE to identify significant factors associated with the correct detection of pneumothorax by the AI. These methods were used to account for data clustering arising from multiple observations per patient.

Diagnostic performance metrics included the sensitivity and specificity of the stand-alone AI and the physicians (in unaided and AI-aided conditions) for pneumothorax detection on supine chest radiographs. Based on the “35-mm rule” [16], performance was evaluated for three categories: (1) overall pneumothorax, (2) large pneumothorax, and (3) small pneumothorax. A large pneumothorax was defined as a maximum radial interpleural distance > 35 mm on the corresponding axial CT image, while a small pneumothorax was defined as ≤ 35 mm. For the stand-alone AI, performance was additionally analyzed by lung zone (upper vs. lower). To account for patient-level clustering, all diagnostic performance metrics were estimated and compared using logistic regression models with GEE.

Statistical significance was set at* P* < 0.05. All statistical analyses were performed using R version 4.5.1 (R Foundation for Statistical Computing, Vienna, Austria; http://www.r-project.org/). The “clusrank” and “geepack” packages were used for the clustered Wilcoxon rank-sum test and GEE-based logistic regression analysis, respectively [23, 24].

## Results

During the study period, 122 pairs of CT scan and supine chest radiograph were obtained from 97 patients with pneumothorax, and 116 pairs from 113 patients without pneumothorax. Table S1 presents the characteristics of the patients with pneumothorax and those without pneumothorax. Of the 97 patients with pneumothorax, 25 (26%) had trauma, and 18 (19%) underwent multiple pairs of CT and supine chest radiography during the study period. Of the 113 patients without pneumothorax, 80 (71%) presented with trauma. Among the 122 CT scans with pneumothorax, slice thicknesses and intervals were 1 mm in 92 (75%), 1.25 mm in 23 (19%), and 5 mm in 7 (6%). In contrast, among the 116 CT scans without pneumothorax, these parameters were 1 mm in 84 (72%), 1.25 mm in 5 (4%), and 5 mm in 27 (23%), respectively. The median time interval between paired examinations with pneumothorax was 52 min (IQR, 14–140 min).

**Fig. 2:**
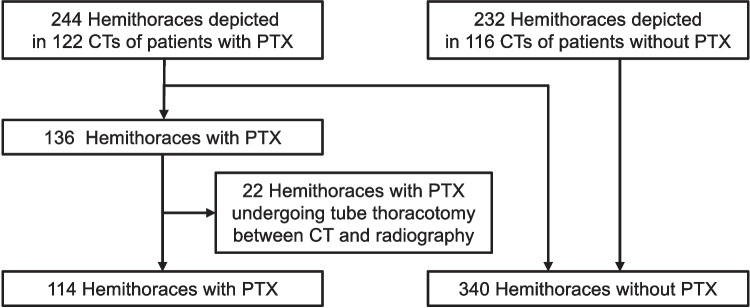
Flow diagram of the reviewed hemithoraces. A total of 114 hemithoraces with pneumothorax and 340 hemithoraces without pneumothorax were ultimately included in the analysis. Twenty-two hemithoraces with pneumothorax were excluded because tube thoracotomy was performed between the paired computed tomography and radiography examinations. PTX, pneumothorax

The 122 CT scans of patients with pneumothorax provided 244 hemithoraces, of which 136 had a pneumothorax, and 108 did not Fig. 2. Of the 136 hemithoraces with pneumothorax, 22 that underwent tube thoracotomy between the paired CT and supine chest radiography were excluded from the subsequent analysis. In contrast, 116 CT scans of patients without pneumothorax provided an additional 232 hemithoraces without pneumothorax. Ultimately, 114 hemithoraces with pneumothorax and 340 hemithoraces without pneumothorax were used for the analysis.

Table 1 presents the characteristics of the pneumothoraces detected by the AI versus those that were missed. The AI correctly identified pneumothoraces in 76 of 114 (67%) hemithoraces. All measured interpleural distances were significantly larger in the pneumothoraces detected by the AI than in those it missed (*P* < 0.001). Among the five measured interpleural distances, the weakest correlation was observed between the apical distance and the basal distance (Spearman’s *ρ* = 0.24). Consequently, these two metrics, along with patient rotation (*P* = 0.05), findings suggestive of chronic lung disease (*P* = 0.005), and trauma (*P* = 0.06), all of which yielded *P* < 0.10 in the univariate analysis, were incorporated into a multivariable logistic regression analysis with a GEE model. The proportion of hemithoraces with a chest tube did not differ significantly between pneumothoraces detected and missed by the AI (45% vs. 42%, *P* = 0.89).

**Table 1:** Comparison of clinical and radiographic characteristics between pneumothoraces detected and missed by the AI

	Overall PTXs (*n* = 114)	PTXs that the AI detected (*n* = 76)	PTXs that the AI missed (*n* = 38)	*P*-value
*Patient demographics*				
Age (years)	64 [50–78]	62 [42–78]	65 [55–80]	0.45
Sex (female)	22 (19)	16 (21)	6 (16)	0.26
*Radiographic positioning*				
Patient rotation	15(13)	7 (9)	8 (21)	0.05
Cranially angulated	19(17)	13 (17)	6 (16)	0.58
Caudally angulated	14(12)	9 (12)	5 (13)	0.43
Side of PTX (left)	52 (46)	33 (43)	19 (50)	0.20
*PTX size (mm)*				
Maximum radial interpleural distance	24 [11–45]	35 [20–70]	13 [7–22]	< 0.001
Apical interpleural distance	6 [0–15]	10 [4–21]	0 [0–5]	< 0.001
Lateral interpleural distance at the midpoint of the upper half of the lung	0 [0–1]	0 [0–2]	0 [0–0]	< 0.001
Lateral interpleural distance at the midpoint of the lower half of the lung	0 [0–4]	0 [0–8]	0 [0–0]	< 0.001
Basal interpleural distance	20 [0–48]	31 [10–53]	0 [0–20]	< 0.001
*CT findings*				
Endotracheal tube	40 (35)	29 (38)	11 (29)	0.82
Chest tube	50 (44)	34 (45)	16 (42)	0.89
Chronic lung disease	38 (33)	31 (41)	7 (18)	0.005
Emphysema	21 (18)	12 (16)	9 (24)	0.49
Interstitial lung disease	11 (10)	10 (13)	1 (3)	0.09
Pneumonia or pulmonary edema	39 (34)	28 (37)	11 (29)	0.46
Pneumomediastinum	11 (10)	8 (11)	3 (8)	0.41
Subcutaneous emphysema	27 (24)	17 (22)	10 (26)	0.65
Trauma	27 (24)	13 (17)	14 (37)	0.06
Post-cardiothoracic surgery	33 (29)	26 (34)	7 (18)	0.17

Data are presented as median [interquartile range] or n (%).

*PTX* Pneumothorax, *CT* Computed tomography.

**Fig. 3:**
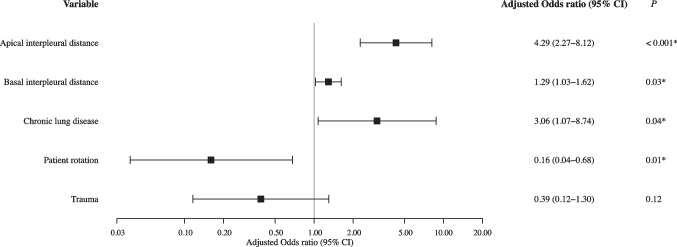
Predictors for correct pneumothorax detection by CXR-AID. Forest plot showing the results of multivariable logistic regression analysis using a generalized estimating equations model to account for data clustering per patient. The adjusted odds ratios for apical and basal interpleural distances are presented per 10-mm increment

Multivariable logistic regression analysis using the GEE model revealed that apical interpleural distance (odds ratio [OR], 4.29; 95% confidence interval [CI]: 2.27–8.12; *P* < 0.001), basal interpleural distance (OR, 1.29; 95% CI: 1.03–1.62; *P* = 0.03), and chronic lung disease (OR, 3.06; 95% CI: 1.07–8.74; *P* = 0.04) were significantly associated with the correct detection of pneumothorax by the AI (Fig. 3). The apical interpleural distance had a significantly stronger association with the correct detection than the basal interpleural distance. In contrast, patient rotation had a significant negative association with the correct detection by the AI (OR, 0.16; 95% CI: 0.04–0.68; *P* = 0.01). Trauma was not significantly associated with detection performance in the multivariable model (*P* = 0.12).

**Fig. 4:**
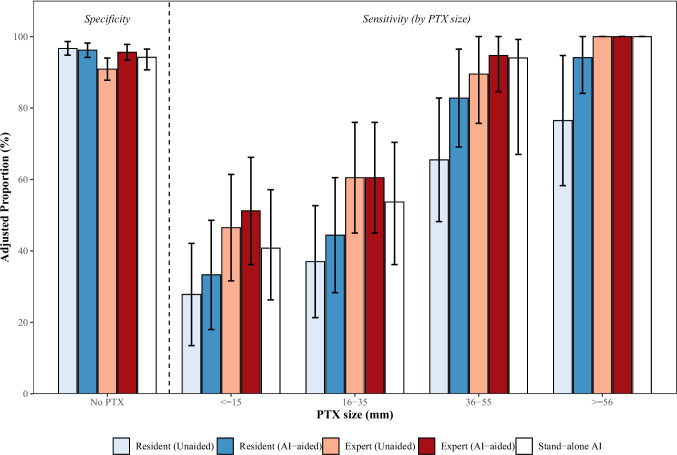
Adjusted specificity and size-dependent sensitivity of stand-alone AI and physicians. Size categories (mm) are based on the maximum radial interpleural distance on axial CT images. Error bars represent 95% confidence intervals. Proportions were adjusted using a generalized estimating equations model to account for data clustering per patient

Figure 4 demonstrates the specificity and size-dependent sensitivity of the stand-alone AI and physicians in the reader study, with and without the AI assistance, in detecting pneumothorax on supine chest radiographs. The stand-alone AI demonstrated an overall adjusted sensitivity of 61.0% (95% CI: 50.6%–70.4%) and a specificity of 94.3% (95% CI: 90.8%–96.5%) (Table S2). For large pneumothoraces (> 35 mm), the adjusted sensitivity was 97.4% (95% CI: 83.6%–99.6%). In contrast, for small pneumothoraces (≤ 35 mm), the adjusted sensitivity was 44.9% (95% CI: 33.3%–57.1%). Of the 114 pneumothoraces, 101 (89%) and 96 (84%) involved the upper and lower lung zones, respectively. The adjusted detection sensitivity for the upper lung zone (69.5%; 95% CI: 59.3%–78.1%) was significantly higher than that for the lower lung zone (37.5%; 95% CI: 27.3%–48.8%). Specificities remained comparable between the upper and lower lung zones. Of the 340 hemithoraces confirmed by CT to be without pneumothorax, 19 (5.6%) were misinterpreted as having pneumothorax by the AI.

**Fig. 5:**
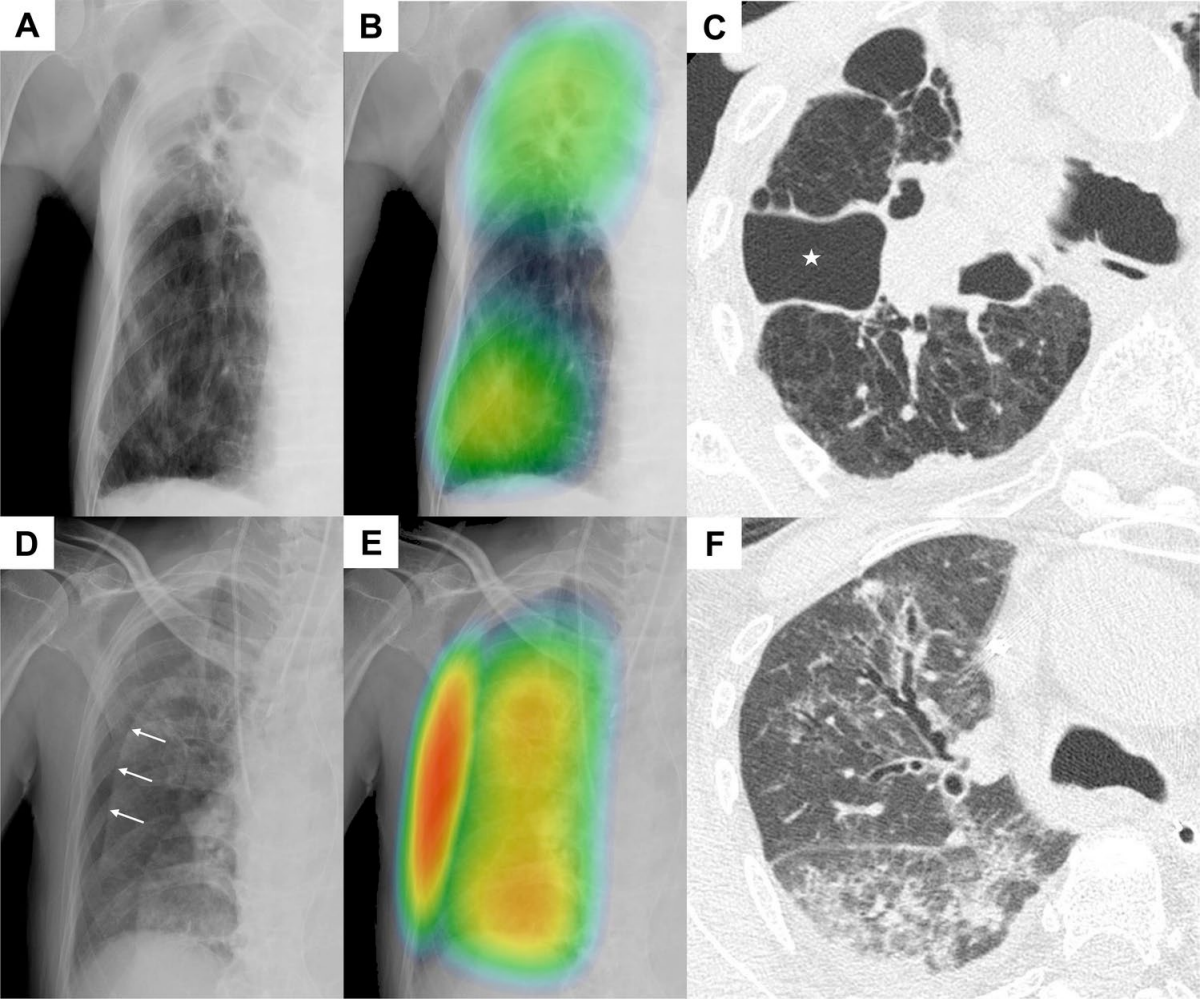
Representative examples of stand-alone AI false-negative and false-positive results. (A–C) A false negative for a large pneumothorax with a maximum radial distance of 38 mm: A case with a loculated pneumothorax in the right upper lung zone. (**A**) Supine chest radiograph showing an opacity in the right upper lung field. (**B**) CXR-AID–generated heatmap highlighting the same region. (**C**) Corresponding axial CT image at the level of the carina. The star indicates the loculated intrapleural air collection. However, the heatmap fails to specifically isolate the air collection from the surrounding opacity. (D–F) A case of a false-positive result in a hemithorax without pneumothorax. (**D**) Supine chest radiograph showing a skin-fold artifact (arrows). (**E**) The CXR-AID–generated heatmap highlights the artifact, which was determined to be a false-positive on expert review with reference to the corresponding CT. (**F**) Corresponding axial CT image confirming the absence of pneumothorax

In the stand-alone analysis, the AI produced one false negative for large pneumothoraces and 19 false positives. The false-negative hemithorax involved a loculated pneumothorax in the right hemithorax with a maximum radial interpleural distance of 38 mm. False-positive hemithoraces included those with skin-fold artifacts (*n* = 10), bullous emphysema (*n* = 4), external medical devices (*n* = 2), subcutaneous emphysema (*n* = 2), and post-cardiothoracic surgery status (*n* = 1). Representative examples of false-negative and false-positive results are demonstrated in Fig. 5.

In the reader study, residents’ sensitivity was significantly higher in the AI-aided condition than in the unaided condition (46.8% vs. 57.3%; *P* < 0.001), whereas no significant difference was observed in experts’ sensitivity (65.6% vs. 68.0%; *P* = 0.32) (Table S3). A similar trend was observed for large pneumothoraces (> 35 mm); AI assistance significantly improved sensitivity for residents (71.4% vs. 88.9%; *P* < 0.001) but did not significantly affect experts' performance (94.4% vs. 97.2%; *P* = 0.31). The AI assistance did not significantly improve sensitivity for small pneumothoraces (≤ 35 mm) in either residents (*P* = 0.14) or experts (*P* = 0.48). Conversely, expert specificity was significantly higher in the AI-aided condition (90.9% vs. 95.6%;* P* = 0.02), while residents showed no significant difference (96.7% vs. 96.2%; *P* = 0.59).

## Discussion

In the present retrospective study, the AI showed favorable sensitivity for detecting pneumothorax on supine chest radiographs, particularly in cases involving large air collections with a maximum radial interpleural distance exceeding 35 mm. In contrast, sensitivity was limited for small pneumothoraces with a maximum radial interpleural distance ≤ 35 mm. Regarding anatomical distribution, the AI’s performance was superior for detecting air collections in the upper lung zones compared to the lower lung zones. Crucially, the reader study suggested that the AI assistance provided distinct, expertise-dependent benefits: it improved sensitivity for residents and specificity for experts in pneumothorax detection.

The present study suggests that, with its high sensitivity in detecting large pneumothorax with a maximum radial interpleural distance > 35 mm, the AI could assist physicians, particularly residents, in ruling out a pneumothorax requiring tube thoracotomy without a CT scan. A related study demonstrated that a patient with traumatic pneumothorax could be safely observed without tube thoracotomy in the presence of a maximum radial interpleural distance ≤ 35 mm on an axial CT image [16]. However, this “35-mm rule” requires the use of a CT scan. The present study suggests that supine chest radiography, when analyzed with the AI, may serve as an alternative to a CT scan in detecting any pneumothorax requiring tube thoracotomy in critically ill patients, for whom transport to a CT room may be hazardous because of clinical instability. However, the sample size for the large pneumothorax subgroup was limited, resulting in wide CIs; therefore, these findings should be interpreted with caution and validated in larger cohorts.

The AI detected air collection in the upper lung zone better than in the lower lung zone. This performance gap is underscored by our multivariable analysis, which established that the apical interpleural distance was more strongly associated with correct pneumothorax detection compared with the basal interpleural distance. Although apical air collection is typically smaller in the supine position than in the upright position, the apical pleural line would still provide a distinct radiographic feature for the AI. In contrast, air in the lower lung zone predominantly accumulates ventrally or medially in the supine position, rather than laterally, resulting in subtle findings that are difficult to detect by the AI on anteroposterior radiographs. Although the deep sulcus sign is a well-recognized radiographic hallmark of pneumothorax on supine radiographs [21, 22], our findings highlight that identifying pneumothorax in the lower lung fields remains a substantial challenge even with AI assistance. Therefore, even when the AI yields a negative result, pneumothorax cannot be excluded, particularly small pneumothoraces with a maximum radial interpleural distance ≤ 35 mm localized to the lower lung zone. Physicians should remain vigilant in patients at risk of pneumothorax progression, such as those receiving positive-pressure ventilation. Further refinement is warranted to enhance the AI’s performance in detecting pneumothorax in the lower lung zone.

The single false-negative case among large pneumothoraces involved a loculated pneumothorax, in which the AI failed to differentiate the air collection from adjacent opacity. This finding suggests that detection may be difficult when pleural adhesions prevent air from extending to the apicolateral regions, where the pleural line is typically visualized on chest radiographs. Among the 19 false-positive cases, skin-fold artifacts likely mimicked the pleural line. Additional false positives occurred in the presence of bullous emphysema, subcutaneous emphysema, external medical devices, and post-surgical changes. Beyond these specific tendencies and limitations, it is important to note that the heatmaps are inherently non-specific. Although the AI is designed to highlight pulmonary nodules, consolidation, and pneumothorax, the physician must determine the specific pathology by correlating the heatmap with the primary radiographic findings. Accordingly, the AI should function strictly as a supportive tool, with final interpretation relying on careful clinical judgment to ensure safe and effective application.

Regarding the AI’s impact on physicians, our study suggests that AI utility is expertise-dependent. For residents, the AI acted as a safety net by significantly increasing sensitivity. In contrast, experts showed no significant sensitivity improvement. This finding is consistent with previous reports in mammography and ultrasound [25, 26]. This lack of improvement is likely attributable to a ceiling effect, as experts’ high baseline sensitivity left limited room for further improvement by the AI. Notably, the AI assistance significantly improved experts’ specificity. This suggests that for experts familiar with subtle radiographic features, the AI reinforces diagnostic confidence, helping to rule out pneumothorax when findings are ambiguous.

The present study evaluated the performance of AI-based software for the detection of pneumothorax on supine chest radiographs, using pneumothorax size measured on corresponding CT images as the reference standard. Previous studies have also developed deep learning-based models for this purpose; however, their performance was evaluated against suboptimal ground truths, such as physician reviews of chest radiographs or size measurements obtained from chest radiographs [12, 27]. In fact, a systematic review showed that the summary sensitivity of supine radiography in detecting pneumothorax was as low as 0.47 (95% CI, 0.31–0.63) [6]. Furthermore, there is no established risk stratification for requiring tube thoracotomy on supine chest radiographs, unlike the “35 mm-rule” on CT images [16].

Ultrasonography showed a higher sensitivity than supine chest radiography in detecting pneumothorax in critically ill patients, as reported in systematic reviews [6, 28]. Nevertheless, the diagnostic performance of ultrasonography could be influenced by the operator's experience and certain patient conditions, such as subcutaneous emphysema, chronic obstructive pulmonary disease, bullous lung disease, pleural adhesions, atelectasis, and low body mass index [28–31]. Thus, it is crucial to improve the diagnostic performance of supine radiography for the detection of pneumothorax to add more information to that obtained using ultrasonography.

Some limitations of the present study should be acknowledged. First, the single-center, single-vendor retrospective design limits the generalizability of our findings. Because performance was evaluated using a specific AI software in a Japanese cohort, the results may not be directly applicable to other AI algorithms or different patient populations. Second, patients who underwent radiography alone were excluded, as radiography and CT were required to establish the reference standard. This inclusion criterion likely enriched the cohort with more severe cases. Consequently, the prevalence of pneumothorax in our cohort may not reflect that of patients undergoing supine chest radiography in routine clinical practice, thereby limiting external validity. Third, we assumed pneumothorax stability between the paired examinations. In clinical practice, any significant expansion of a pneumothorax after radiographic or CT confirmation would necessitate immediate tube thoracotomy. Since we excluded all cases requiring such interventions between the two scans, we inferred that the pneumothorax volumes remained relatively constant. Nevertheless, the median 52-min interval may have allowed minor progression or partial resolution, representing a potential limitation in the strict correlation between CT findings and radiographic assessment. In addition, although physicians visually confirmed correspondence between heatmaps and pneumothorax locations using CT as a reference, we did not perform a quantitative spatial analysis using objective metrics. Fourth, a chest tube was present in 44% of hemithoraces with pneumothorax in the primary analysis. Previous studies have reported that the presence of a chest tube can affect the performance of some AI algorithms in detecting pneumothorax on chest radiographs [14, 32]. Nevertheless, there were no significant differences in the proportion of patients with chest tubes between pneumothoraces detected by the AI and those that the AI missed. This suggests that the presence of a chest tube is not a significant confounding factor for the AI performance. Fifth, although the reader study demonstrated improved diagnostic performance with AI assistance, we did not evaluate actual changes in clinical management or patient outcomes. Therefore, the clinical impact of these findings remains theoretical. Prospective studies are warranted to determine whether AI assistance improves decision-making and patient outcomes in real-world practice. Finally, the reader study involved a limited number of participants (three residents and three experts), all from a single institution, which may not fully represent the diversity of diagnostic performance across all clinical settings.

## Conclusion

The present study suggests that CXR-AID, an AI-based software, facilitates the detection of pneumothorax on supine chest radiographs, particularly large air collections requiring tube thoracotomy. The benefit of AI assistance appeared to be expertise-dependent: it functioned as a diagnostic safety net for residents by significantly increasing sensitivity, while enhancing diagnostic confidence among experts by improving specificity. However, detection of intrapleural air collections in the lower lung zone remains challenging. Future multicenter studies are warranted to validate these findings, and further refinement of AI algorithms is required to improve performance in detecting pneumothorax in the lower lung zone.

## Supplementary Information

Below is the link to the electronic supplementary material.

Supplementary file1 (PDF 100 KB)

## Data Availability

The datasets generated during and/or analyzed during the current study are available from the corresponding author on reasonable request, subject to permission from the Institutional Review Board of the University of Tokyo Hospital.
