# Supplementary material for: Performance of an artificial intelligence–based software in detecting pneumothorax on supine chest radiographs: a retrospective study
Source: Emerg Radiol. 2026 Mar 11;33(2):281–90. doi: 10.1007/s10140-026-02448-4 (PMC13079510; doi:10.1007/s10140-026-02448-4)
Supplement: Supplementary file 1 — Supplementary file1 (PDF 100 KB) [file 10140_2026_2448_MOESM1_ESM.pdf]

Supplemental materials

Table S1. Characteristics of patients with and without pneumothorax

	With PTX (n = 97)	Without PTX (n = 113)
Age (years)	65 [52–79]	59 [40–76]
Sex (female)	21 (22)	40 (34)
Comorbidities		
Emphysema	26 (27)	26 (23)
Interstitial lung disease	10 (10)	4 (4)
Trauma	25 (26)	80 (71)
Number of computed tomography–radiography pairs per patient		
1	79 (81)	110 (97)
2	13 (13)	3 (3)
3	4 (4)	0 (0)
5	1 (1)	0 (0)

Data are presented as median [interquartile range] or n (%).

PTX, pneumothorax

Table S2. Stand-alone AI performance in detecting intrapleural air on supine chest radiographs

	Sensitivity (%)		Specificity (%)	
	Unadjusted	Adjusted (95% CI) *	Unadjusted	Adjusted (95% CI) *
Overall PTX	66.7 (76/114)	61.0 (50.6–70.4)	94.4 (321/340)	94.3 (90.8–96.5)
Size of intrapleural air				
Large PTX †	97.4 (38/39)	97.4 (83.6–99.6)	NA	NA
Small PTX †	50.7 (38/75)	44.9 (33.3–57.1)	NA	NA
Location of intrapleural air				
Upper lung zone	70.3 (71/101)	69.5 (59.3–78.1)	95.8 (338/353)	95.9 (92.6–97.7)
Lower lung zone	41.7 (40/96)	37.5 (27.3–48.8)	97.2 (348/358)	97.3 (94.6–98.7)

* Adjusted values account for data clustering arising from multiple observations per patient with generalized estimating equations.

† Large PTX and small PTX refer to pneumothorax where the maximum radial interpleural distance on an axial CT image is >35 mm and ≤35 mm, respectively.

CI, confidence interval; NA, not applicable; PTX, pneumothorax

Table S3. Diagnostic performance of physicians under unaided and AI-aided conditions

	Unaided (%)	Aided (%)	Difference (%)	(95% CI) *	*P*-value
All physicians					
Sensitivity for overall PTX	54.6	61.8	7.2	(3.0 to 11.3)	<0.001
Sensitivity for large PTX †	79.8	91.9	12.1	(6.9 to 17.4)	<0.001
Sensitivity for small PTX †	41.8	46.4	4.6	(−0.8 to 10.1)	0.10
Specificity	94.4	96.0	1.6	(−0.5 to 3.7)	0.13
Residents					
Sensitivity for overall PTX	46.8	57.3	10.5	(4.4 to 16.6)	<0.001
Sensitivity for large PTX †	71.4	88.9	17.5	(9.8 to 25.1)	<0.001
Sensitivity for small PTX †	32.4	38.9	6.5	(−2.1 to 15.1)	0.14
Specificity	96.7	96.2	−0.5	(−2.5 to 1.5)	0.59
Experts					
Sensitivity for overall PTX	65.6	68.0	2.5	(−2.4 to 7.3)	0.32
Sensitivity for large PTX †	94.4	97.2	2.8	(−2.6 to 8.2)	0.31
Sensitivity for small PTX †	53.5	55.8	2.3	(−4.2 to 8.9)	0.48
Specificity	90.9	95.6	4.8	(0.9 to 8.7)	0.02

* The 95% confidence intervals were estimated using generalized estimating equations to account for clustering within patients.

† Large PTX and small PTX refer to pneumothorax where the maximum radial interpleural distance on an axial CT image is >35 mm and ≤35 mm, respectively.
